# Exogenous strigolactone interacts with abscisic acid-mediated accumulation of anthocyanins in grapevine berries

**DOI:** 10.1093/jxb/ery033

**Published:** 2018-01-31

**Authors:** Manuela Ferrero, Chiara Pagliarani, Ondřej Novák, Alessandra Ferrandino, Francesca Cardinale, Ivan Visentin, Andrea Schubert

**Affiliations:** 1 PlantStressLab, Department of Agricultural, Forestry, and Food Sciences, University of Turin, Grugliasco, Italy; 2 Laboratory of Growth Regulators, Palacký University & Institute of Experimental Botany AS CR, Olomouc, Czech Republic

**Keywords:** ABA conjugation, ABA hydroxylases, ABA transporters, abscisic acid, anthocyanin, grapevine, GR24, ripening, strigolactones

## Abstract

Besides signalling to soil organisms, strigolactones (SLs) control above- and below-ground morphology, in particular shoot branching. Furthermore, SLs interact with stress responses, possibly thanks to a crosstalk with the abscisic acid (ABA) signal. In grapevine (*Vitis vinifera* L.), ABA drives the accumulation of anthocyanins over the ripening season. In this study, we investigated the effects of treatment with a synthetic strigolactone analogue, GR24, on anthocyanin accumulation in grape berries, in the presence or absence of exogenous ABA treatment. Experiments were performed both on severed, incubated berries, and on berries attached to the vine. Furthermore, we analysed the corresponding transcript concentrations of genes involved in anthocyanin biosynthesis, and in ABA biosynthesis, metabolism, and membrane transport. During the experiment time courses, berries showed the expected increase in soluble sugars and anthocyanins. GR24 treatment had no or little effect on anthocyanin accumulation, or on gene expression levels. Exogenous ABA treatment activated soluble sugar and anthocyanin accumulation, and enhanced expression of anthocyanin and ABA biosynthetic genes, and that of genes involved in ABA hydroxylation and membrane transport. Co-treatment of GR24 with ABA delayed anthocyanin accumulation, decreased expression of anthocyanin biosynthetic genes, and negatively affected ABA concentration. GR24 also enhanced the ABA-induced activation of ABA hydroxylase genes, while it down-regulated the ABA-induced activation of ABA transport genes. Our results show that GR24 affects the ABA-induced activation of anthocyanin biosynthesis in this non-climacteric fruit. We discuss possible mechanisms underlying this effect, and the potential role of SLs in ripening of non-ABA-treated berries.

## Teaser

The strigolactone analogue GR24 reduces ABA-induced anthocyanin accumulation in *Vitis vinifera* berries. GR24 treatment does not affect ABA biosynthesis while it activates ABA degradation and possibly ABA membrane transport.

## Introduction

Grapevine ranks fourth among major fruit crops worldwide, and first in Europe (http://www.fao.org/faostat/en/#data). Ripe berries are employed for direct consumption and for wine production. At harvest, an optimal balance among berry components (sugars, acids, and secondary metabolites) is an absolute requirement to guarantee consumer preference and commercial success. Grape berry secondary metabolites are represented by many polyphenols (Adams, 2006) and volatile compounds (Kalua and Boss, 2010). Overall, these molecules contribute to the colour, taste, and aroma of grapes, and are involved in wine stabilization and ageing. Anthocyanins are one of the major groups of polyphenols in berry skins of coloured cultivars. Their concentration and diversity control colour intensity and stability in the fruit and in the wine derived therefrom; furthermore, they contribute to seed dispersal and defence against oxidative stress. Anthocyanins are absent in the first stage of berry development, while they accumulate in vacuoles from the start of berry ripening (véraison) (Moskowitz and Hrazdina, 1981).

The molecular and physiological processes controlling ripening and anthocyanin accumulation in the non-climacteric grape berry are still poorly known, although great strides forward have been made, in particular through the application of transcriptomic (Deluc *et al.*, 2007) and proteomic (Giribaldi *et al.*, 2007) approaches. Hormonal control of fruit ripening is a well-described process, and several hormones were shown to interact with some aspects of ripening in grape. Auxins, brassinosteroids, and salicylic acid have an inhibitory effect on berry ripening (Davies *et al.*, 1997; Symons *et al.*, 2006). Disruption of ethylene perception negatively affects anthocyanin accumulation (Chervin *et al.*, 2004), but the relevance of ethylene in berry ripening is debated (Sun *et al.*, 2010). Methyl jasmonate treatments enhance anthocyanin accumulation in suspension cultures (Belhadj *et al.*, 2008) and in whole berries (Symons *et al.*, 2006; Jia *et al.*, 2016). Besides these hormones, abscisic acid (ABA) has been long suspected to be the master controller of ripening in grapevine, as both its biosynthesis (Deluc *et al.*, 2007) and concentration in the berry (Coombe and Hale, 1973; Davies *et al.*, 1997) peak at véraison. This hypothesis is further supported by observation that exogenous ABA activates accumulation of anthocyanins and sugars in the grape berry (Coombe and Hale, 1973; Wheeler *et al.*, 2009), and expression activation of anthocyanin biosynthetic genes and of transcription factors controlling this pathway (Jeong *et al.*, 2004; Gambetta *et al.*, 2010; Giribaldi *et al.*, 2010; Villalobos-González *et al.*, 2016). The role of ABA in the induction of anthocyanin accumulation is not limited to the grape berry, indeed it has been demonstrated in other non-climacteric fruits (Kadomura-Ishikawa *et al.*, 2015) and in Arabidopsis and maize seed vegetative tissues (McCarty *et al.*, 1989).

Strigolactones (SLs) were first discovered for their ability to induce seed germination of root parasite plants when exuded in soil (Bradow and Connick, 1988). Later on, they were demonstrated to play an essential role as plant signals for other soil organisms, such as arbuscular mycorrhizal (AM) fungi (Akiyama *et al.*, 2005) and symbiotic nitrogen-fixing bacteria (Peláez-Vico *et al.*, 2016). The study of Arabidopsis and rice branching mutants showed, however, that SLs also strongly repress the growth of axillary buds (Gomez-Roldan *et al.*, 2008; Umehara *et al.*, 2008). The action of SL on shoot branching may be mediated by complex interaction with other hormones, namely auxin and cytokinins (Ruyter-Spira *et al.*, 2013).

SL concentration is responsive to nutrient deprivation, in particular of phosphorus and nitrogen (Yoneyama *et al.*, 2007). This is seen as an adaptive strategy to regulate interaction with AM fungi: plants increase SL production under nutrient starvation, in order to minimize shoot branching and promote AM colonization (Gomez-Roldan *et al.*, 2008; Umehara *et al.*, 2008). Recent studies have demonstrated that SLs are also involved in responses to other abiotic stresses, in particular drought. Arabidopsis, Lotus, and tomato genotypes with reduced SL levels are hypersensitive to drought stress (Ha *et al.*, 2014; Liu *et al.*, 2015; Visentin *et al.*, 2016; Lv *et al.*, 2018), while SL supplementation abolishes the drought-sensitive genotype. In most of these studies, SL-dependent changes in stress susceptibility were mainly linked to an ABA signalling-dependent modulation of stomatal closure, suggesting that SLs may interact with the ABA signal upon stress. These observations raise the question of whether SLs can also interact with ABA in developmentally regulated processes, such as ripening of the non-climacteric grape berries.

In this study, we investigated the effect of modifications of exogenous SL on ABA-induced ripening of grapevine berries. By application of the SL analogue GR24 (Besserer *et al.*, 2008) to berries at véraison in the presence and absence of exogenous ABA, we demonstrate that exogenous SL down-regulates the effects of exogenous (but not endogenous) ABA, possibly by affecting its metabolism and transport.

## Materials and methods

### Plant material and experimental set-up

Experiments were performed on *Vitis vinifera* cultivar Barbera, whose anthocyanin profile is dominated by mono- and di-methylated forms (Ferrandino *et al.*, 2012).

Treatments were applied in a first experiment on detached, *in vitro* incubated berries. This technique has often been used to study ripening processes in grape; however, the berries at this stage are exchanging substances with the plant via the vascular system and, to take this into account, we replicated our treatments in a second experiment on intact berries attached to the plant.

For the *in vitro* experiment, non-coloured, field-grown berries were collected at the start of ripening (véraison) 2015 from vines at the Grugliasco campus vineyard (Piedmont, Italy, 45°03'55''N, 7°35'35''E) by severing the apical end of their pedicel. Vines were trellised and Guyot-pruned, subjected to standard management techniques, and véraison started on 22 July 2015 (52 d after flowering). Berries were surface-sterilized with 70% ethanol followed by a 20% (w/v) NaClO solution, then rinsed with sterile water. Berries were laid in sterile Petri dishes (~10 berries per dish) in close contact (on the petiole side) with agar containing 8% (w/v) sucrose and the following combinations of ±ABA (Sigma) and *rac*-GR24 (Strigolab, Turin, Italy): no hormones; ±ABA 200 µM; *rac*-GR24 10^−5^ M; ±ABA 200 µM; and *rac*-GR24 10^−5^ M. To prevent contamination, the whole procedure was conducted under sterile conditions in a laminar hood. Sixty berries per treatment were collected 0, 24, 72, and 144 h after the start of the experiment, frozen in liquid nitrogen, and stored at –80 °C.

For the experiment on attached berries, grape bunches from 10 vines were sprayed once at the start of véraison until run-off, in the late afternoon and with the same hormone combinations, omitting sucrose (two bunches per treatment, each from a different vine). During the period of treatment, bunches were protected from direct sunlight by shading nets. Sixty berries per treatment were collected 0, 48, and 144 h after spraying, by severing the apical end of the pedicel. Berries were frozen in liquid nitrogen, and stored at –80 °C.

Additional samples of non-treated berries were taken at different stages of development to assess expression of SL biosynthetic genes.

Frozen berries were quickly peeled, and berry skins were powdered in liquid nitrogen and stored at –80 °C until analysis, while flesh was used for measurement of soluble solids.

### Soluble sugars, total anthocyanin, and ABA concentration

Soluble sugars were assessed in triplicate with a refractometer on 10 extracts of berry flesh obtained by pressing.

Anthocyanin content was quantified in triplicate on ~1.5 g of powdered skin tissue, diluted 1:10 with acidic ethanol chloride (CH_3_CH_2_OH:H_2_O:HCl 70:30:1 v/v/v), by spectrophotometric analysis, reading the absorbance at 520 nm (Ferrandino and Guidoni, 2010).

ABA was quantified by LC-MS (Floková *et al.*, 2014). A 15 mg sample from powdered berry skins was extracted using 1 ml of cold extraction solvent (10% methanol). In the same tube, 10 µl of stable isotope-labelled standard (D6-ABA 10^−6^ M) were added together with ceramic beads, in order to facilitate the homogenization with a Tissue Lyser (Qiagen) for 5 min at 27 Hz. The homogenates were sonicated for 3 min at 4 °C and shaken for 30 min at 4 °C. Samples were then centrifuged for 15 min at 20 000 rpm (4 °C). The supernatant was filtered using Oasis HLB extraction cartridges (30 μm cut-off) previously conditioned with 2 ml of 100% CH_3_OH and 1 ml of redistilled water. For the elution, 3 ml of 80% CH_3_OH were used, evaporated to dryness under a gentle stream of nitrogen at 30 °C for ~2 h. The dried residue was resuspended in 40 ml of 15% acetonitrile+85% HCOOH, filtered using 2 ml filtration tubes of 0.2 µm, and analysed with an Acquity UPLC^®^ system (Waters, Milford, MA, USA) coupled to a quadrupole mass spectrometer Xevo™ TQ MS (Waters MS Technologies, Manchester, UK). Each sample (10 µl) was first separated on an RP column (Acquity^®^ UPLC CSH™ C18; 1.7 µm, 2.1 × 100 mm) at a flow rate of 0.4 ml min^−1^, using the following solvents: 10 mM HCOOH (A) and acetonitrile (B). The gradient elution over 35 min was as follows: 0–5 min isocratic elution (15% A; v/v); 5–15 min linear gradient to 45% A; 15–28 min, logarithmic gradient to 48.6% A; 28–29 min linear gradient to 100% A. Finally, the column was washed with 100% acetonitrile and then equilibrated to the initial conditions (15% A, v/v) for 5 min. The effluent was introduced into the ESI (electrospray ionization) ion source of a tandem MS analyser with a cone/desolvation gas temperature of 120/550 °C at a flow of 70/650 l h^−1^, with the capillary voltage set to 3 kV; cone voltage, 23–30 V; collision energy, 12–23 eV; and collision gas flow (argon), 0.21 ml min^−1^. Detection was performed by multiple reaction monitoring (MRM) in positive ion mode. Optimization of fragmentation was done with labelled standards using the MAssLynx™ software package (version 4.1, Waters).

Matrix effects were calculated as the ratio of the mean peak area of the analyte spiked post-extraction to the mean peak area of the same analyte standards multiplied by 100. The process efficiency was determined as the mean peak area of the added standards before sample preparation divided by the known mean peak area of standard solutions. For assessment of the validation method, the concentration of the analyte was calculated using the standard isotope dilution method for each plant extract spiked before extraction and compared with the concentration of a proper standard solution. Each measurement was performed in quadruplicate.

### *In silico* and quantitative reverse transcription–PCR analysis

Two putative biosynthetic genes for SL, namely the *Carotenoid Cleavage Dioxygenases* (*CCD*) *7* and *8*, were identified by BLAST searching the grapevine ‘PN40024’ 12X genome draft, V1 annotation, at the Grape Genome Database (http://genomes.cribi.unipd.it/grape/) with the Arabidopsis sequences.

Concentration changes of target transcripts were quantified on powdered berry skin samples (1.5 g) by quantitative reverse transcription–PCR (RT–qPCR). Total RNA was extracted following a cetyltrimethylammonium bromide (CTAB)-based protocol (Carra *et al.*, 2007). RNA integrity and quantity were checked using a 2100 Bioanalyzer (Agilent Technologies). RNA samples were treated with RNase-free DNase I (Fermentas: 50 U µl^−1^ UAB, Vilnius, Lithuania) to avoid any risk of genomic DNA contamination, and first-strand cDNA was synthesized starting from 5 µg of total RNA using the High Capacity cDNA Reverse Transcription kit (Applied Biosystems, Foster City, CA, USA) following the manufacturer’s instructions. cDNA integrity and primer specificity were then checked by gradient PCR and agarose gel electrophoresis. RT–qPCR was conducted in triplicate using a StepOnePlus™ System (Applied Biosystems), and the SYBR Green method (Power SYBR^®^ Green PCR Master Mix, Applied Biosystems) was used for quantifying amplification results (Giordano *et al.*, 2016; Pagliarani *et al.*, 2017). Each reaction contained 1 μl of 5 μM primer mix, 100 ng of template cDNA, 5 μl of 2× SYBR Green mix, and 3 μl of diethylpyrocarbonate (DEPC)-treated water for a total reaction volume of 10 μl. Thermal cycling conditions were as follows: 95 °C for 10 min before the beginning of the amplification (holding stage), followed by 40 cycles at 95 °C for 15 s and 60 °C for 1 min. Specific annealing of primers was further checked on dissociation kinetics at the end of each RT–qPCR run. Expression of target transcripts was quantified after normalization to the geometric mean of the *Ubiquitin* (*VvUBI*) and *Actin* (*VvACT1*) transcripts used as endogenous controls. Expression changes were analysed for *VvMybA1* (encoding a Myb transcription factor controlling anthocyanin biosynthesis in grapevine: Walker *et al.*, 2007), *VvUFGT* (terminal gene of anthocyanin biosynthesis in grapevine, encoding UDP-glucose:flavonoid 3-*O*-glucosyltransferase: Ford *et al.*, 1998), *VvNCED1* (rate-limiting gene of ABA biosynthesis, encoding 9-*cis*-epoxycarotenoid dioxygenase: Wheeler *et al.*, 2009), two genes encoding ABA 8'-hydroxylases (*VvHYD1* and *VvHYD2*; Speirs *et al.*, 2013), an ABA-UDPG glycosyl transferase (*VvGT1*; Sun *et al.*, 2015), a β-glucosidase that hydrolyses ABA-glucose ester (*VvBG1*; Sun *et al.*, 2015), and the grapevine orthologues of the Arabidopsis ABC Transporter G Family Protein (ABCG) ABA membrane transporters *VvABCG25* (Kuromori *et al.*, 2010) and *VvABCG40* (Kang *et al.*, 2010). Transcript quantification of the putative grapevine *CCD7* and *CCD8* was performed on non-treated berry samples only. Gene-specific primer pairs used in RT–qPCR experiments are listed in Supplementary Table S1 at *JXB* online.

### Statistical analyses

For all measurements, three replicates of 10 berries were extracted and analysed independently for each treatment and sampling time. Significant differences among treatments were statistically evaluated by applying a one-way ANOVA using the Tukey’s HSD post-hoc test for separating means when ANOVA results were significant (*P*<0.05). The SPSS statistical software package was used for the analysis (SPSS Inc., Cary, NC, USA, v.22).

## Results

### Ripening and colour turning

**Fig. 1.**
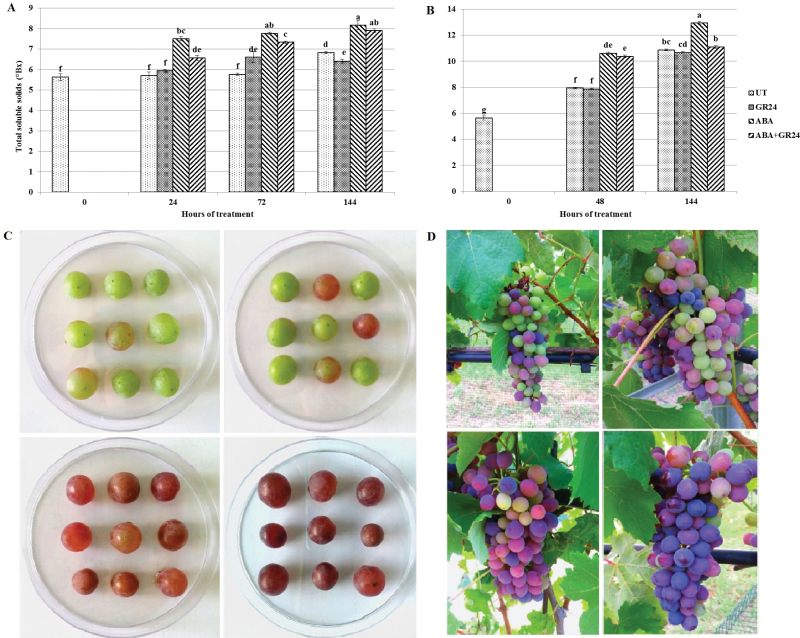
Accumulation of soluble solids (A, B) and colour turning (C, D) in *V. vinifera* berries (A, C) severed from the vine and incubated at véraison in the presence of different hormones, or (B, D) attached to the vine and sprayed at véraison with the same hormone combinations. UT, untreated control (no hormones); GR24, *rac*-GR24 10^−5^ M; ABA, ±ABA 200 µM; ABA+GR24, *rac*-GR24 10^−5^ M and ABA 200 µM. (C and D) Pictures were taken 6 d after treatment; treatments are displayed clockwise starting from the upper left panel. Values marked by the same letter do not significantly differ at *P*=0.05; bars are SEs.

In order to investigate both specific and combined effects of GR24 and ABA on ripening of grape berries, we incubated detached berries *in vitro* on medium supplied with sucrose and hormones. Furthermore, in a second experiment, the same hormone treatments were applied to intact berries at véraison, to avoid the possible interference by exogenous sucrose and to allow for transport processes to the berry via the intact vasculature. Ripening, as shown by the accumulation of soluble sugars, proceeded as expected in untreated berries, in particular in those attached to the plant that were able to import phloematic sugar. Accumulation of soluble solids was slightly (but not significantly) hampered by GR24; it was significantly enhanced by exogenous ABA; however, this effect was counteracted by GR24 co-treatment (Fig. 1A, B). Also in both experiments, ABA induced colour turning; effects of treatment with GR24 in the absence of ABA were not visible, while GR24 administered together with ABA delayed colour accumulation compared with the samples treated with ABA alone (Fig 1C, D).

### Anthocyanin accumulation

**Fig 2.**
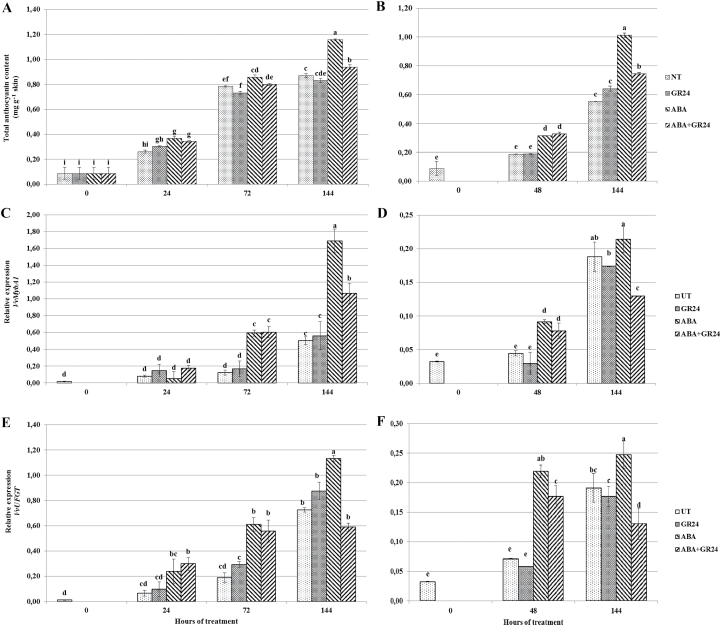
Anthocyanin accumulation (A, B) and transcript accumulation of regulatory (*VvMybA1*: C, D) and biosynthetic (*VvUFGT*: E, F) genes of anthocyanin biosynthesis in *V. vinifera* berry skins (A, C, E) severed from the vine and incubated at véraison in the presence of different hormones, or (B, D, F) attached to the vine and sprayed at véraison with the same hormone combinations. For treatment labels and significance of differences, see legend to Fig. 1.

Colour changes were reflected in anthocyanin concentrations, which increased above those of untreated control following ABA treatment from the first sampling time onwards in both experiments. When berries were treated with GR24 only, the anthocyanin concentration was in some cases slightly lower, but never differed significantly from that measured in untreated control samples. When combined ABA and GR24 were supplied to the medium, anthocyanin accumulation was significantly lower than in the case of berries treated with ABA alone; this trend was observed in both experiments, and was particularly evident at the end of the time course (Fig. 2A, B).

The transcript concentrations of *VvMybA1* (Fig 2B, C) and *VvUFGT* (Fig 2E, F) followed the pattern of anthocyanin accumulation well. In untreated controls, transcripts progressively accumulated to reach significantly higher amounts at the end of the experiment. In berries treated with GR24, transcript levels of these genes showed no difference from untreated controls at the same sampling times. In ABA-treated berries, the concentration of *VvMybA1* and *VvUFGT* transcripts underwent a significant increase above that of the untreated control from 48 h (*in vitro*) or 72 h after treatment (in intact berries), confirming that expression of these genes is induced by exogenous ABA. The combined application of ABA and GR24 negatively affected the expression of both genes compared with treatment with ABA alone, in most cases limiting transcript accumulation to the level observed in untreated berries.

### ABA concentration and biosynthesis

**Fig. 3.**
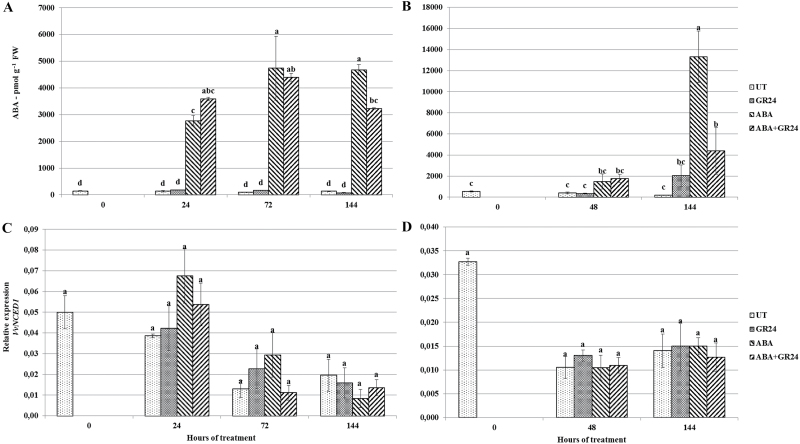
ABA concentration (A, B) and transcript accumulation of the ABA biosynthetic gene *VvNCED1* (C, D) in *V. vinifera* berry skins (A, C) incubated at véraison in the presence of different hormones, or (B, D) attached to the vine and sprayed at véraison with the same hormone combinations. For treatment labels and significance of differences, see legend to Fig. 1.

We explored whether GR24 could act on the anthocyanin concentration by modulating ABA concentrations. ABA levels showed no significant changes over time in the untreated control samples; average concentrations across all sampling times were significantly higher in attached than in *in vitro* incubated berries (391 pmol g FW versus 125 pmol g−1 FW), consistent with ABA phloematic transport to the berry (Fig. 3A, B). No significant effects of treatment with GR24 alone were detected. As expected, in ABA-treated berry skins, ABA concentration drastically increased at the first sampling time and remained stable in incubated berries (Fig. 3A), while the increase was slower in attached berries (Fig. 3B). GR24 co-treatment induced no significant effects on ABA skin concentration in the intermediate measurements, while at the end of both experiments these berries contained significantly less ABA than berries treated with ABA alone (Fig. 3A, B).

The expression trend of the ABA biosynthetic gene *VvNCED1* featured a decline in transcript levels over time in both experiments, and was not affected by treatment with the different hormone combinations (Fig. 3C, D).

### ABA metabolism and transport

**Fig. 4.**
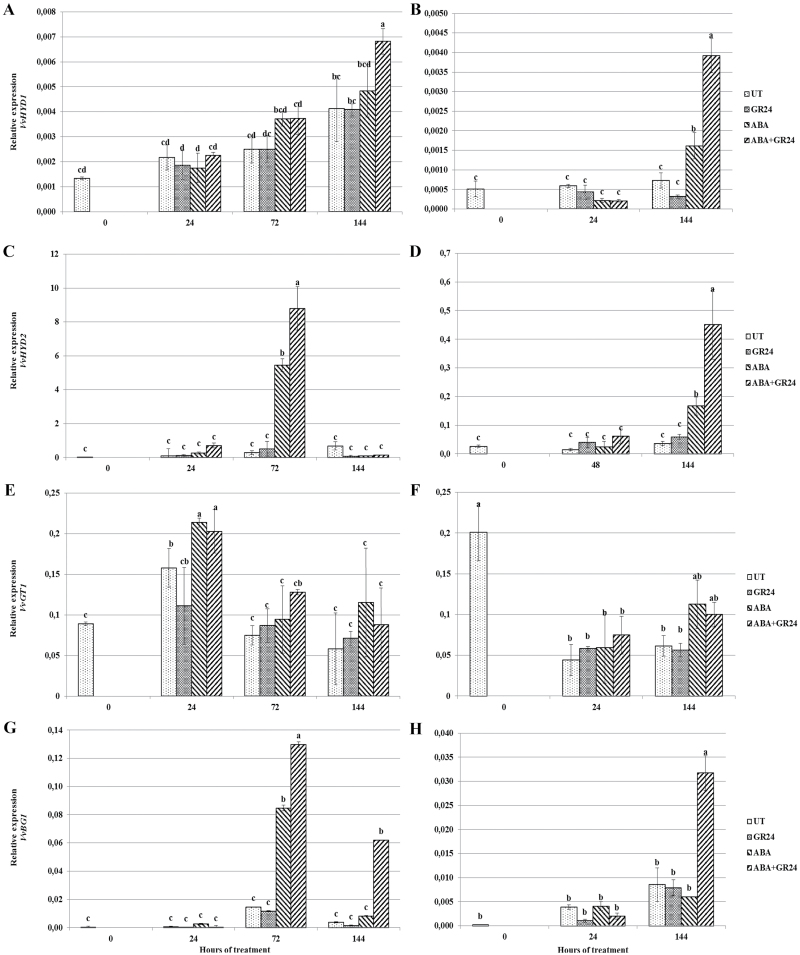
Transcript accumulation of genes involved in ABA metabolism. Relative expression of *VvHYD1* (A, B), *VvHYD2* (C, D), *VvGT1* (E, F), and *VvBG1* (G, H) in *V. vinifera* berry skins (A, C, E, G) incubated at véraison in the presence of different hormones, or (B, D, F, H) attached to the vine and sprayed at véraison with the same hormone combinations. For treatment labels and significance of differences, see legend to Fig. 1.

The effect of exogenous GR24 on ABA metabolism was further explored by analysing the expression of genes involved in ABA hydroxylation (*VvHYD1* and *VvHYD2*), conjugation (*VvGT1*), and de-conjugation (*VvBG1*). Expression of *VvHYD1* increased during both time courses, and was significantly higher at the end of the experiment in attached ABA-treated berries than in untreated controls, and even significantly higher following co-treatment with the two hormones in both experiments (Fig. 4A, B). Similar transcript profiles were observed for *VvHYD2* in attached berries (Fig 4C, D), while *in vitro* the concentration peak was anticipated at 72 h after the start of the experiment. Expression of *VvGT1* did not significantly differ among treatments at each sampling time (Fig 4E, F). Transcript accumulation of *VvBG1* was enhanced by ABA only in incubated berries at 72 h from the beginning of the experiment, whereas ABA+GR24 co-treatment consistently and significantly increased expression in both experiments (Fig 4H, G).

**Fig. 5.**
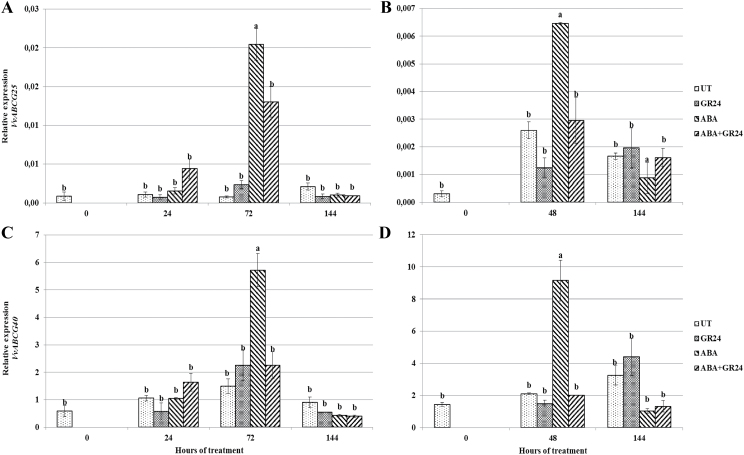
Transcript accumulation of genes involved in ABA transport. Relative expression of *VvABC25* (A, B) and of *VvABCG40* (C, D) in *V. vinifera* berry skins (A, C) incubated at véraison in the presence of different hormones, or (B, D) attached to the vine and sprayed at véraison with the same hormone combinations. For treatment labels and significance of differences, see legend to Fig. 1.

ABA transporters tune the level of cytosolic ABA and thus the responses due to ABA recognition by PYR-like/Regulatory Component of ABA Receptor (PYL/RCAR) cytosolic receptors. The transcript levels of the putative ABA transporter-encoding genes *VvABCG25* (Fig 5A, B) and *VvABCG40* (Fig 5C, D) were thus monitored, showing no significant concentration changes in either untreated or GR24-treated berries throughout the experiments. In contrast, transcript levels of these genes increased significantly following ABA treatment, peaking at 72 h and 48 h in the berries treated with ABA *in vitro* and *in vivo*, respectively, and decreasing afterwards. Co-treatment with GR24 and ABA significantly limited this increase or hindered it completely.

## Discussion

### Exogenous SL negatively interacts with ABA-induced anthocyanin accumulation in grape berries

Accumulation of soluble sugars and, in coloured varieties, of anthocyanins are main facets of grape berry ripening. Grape berries contain glucose and fructose as soluble sugars, and glucosides of cyanidin, delphinidin, peonidin, petunidin, and malvidin, the latter predominant in the majority of coloured cultivars, such as Barbera (Ferrandino *et al.*, 2012). Total soluble sugar content increases from about 5°Brix at véraison (start of ripening) to well above 20°Brix at the end of ripening; anthocyanins accumulate from véraison during 20–40 d (Hrazdina *et al.*, 1984) to reach final concentrations >1.2 mg g^−1^ in skin tissue in Barbera (Ferrandino *et al.*, 2012).

Exogenous ABA supplemented via the severed pedicel or sprayed on intact grape berries enhances sugar content and anthocyanin accumulation (Pirie and Mullins, 1976; Wheeler *et al.*, 2009; Sandhu *et al.*, 2011). In both our experiments, ABA-treated berries followed this pattern, and reacted to exogenous ABA with an increase in soluble sugars and anthocyanins. Some molecular markers of anthocyanin accumulation are well known in grape berries: expression of the MYB transcription factor *VvMybA1*, encoding a transcriptional regulator that activates anthocyanin biosynthesis (Walker *et al.*, 2007), and of the *UDP-glucose:flavonoid 3-O-glucosyltransferase* (*VvUFGT*) gene, encoding the last step of the anthocyanin biosynthetic pathway (Ford *et al.*, 1998), closely follows the pattern of anthocyanin accumulation, and they are correspondingly activated by exogenous ABA (Jeong *et al.*, 2004), as confirmed in our experiments.

The main finding of this study is that GR24 modified this pattern as it markedly inhibited the ABA-induced accumulation of both sugars and anthocyanins, and the transcriptional increase of *VvMybA1* and *VvUFGT*. GR24 is a synthetic SL analogue widely used to simulate the action of natural compounds, also due to its ability to permeate plant tissues, as shown by the fact that it efficiently reverts the effects of genetic SL depletion (Ruyter-Spira *et al.*, 2011; Visentin *et al.*, 2016; Ito *et al.*, 2017), and that it can be detected within treated tissues (Liu *et al.*, 2015). We thus assume that GR24 concentration increased in GR24-treated berries, as was the case for ABA following ABA treatment.

The effects of GR24 were accompanied by a significant reduction of ABA concentration in ABA-treated berries, compared with those treated with ABA only, suggesting that the effects of GR24 were mediated by changes in the ABA signal. Bi-directional hormone interactions involving ABA and SL have been reported in other experimental systems. In tomato, chemically or genetically induced reduction of ABA concentration inhibits SL biosynthesis (López-Ráez *et al.*, 2010). Conversely, changes in SL levels or sensitivity affect ABA concentration and responses: SL-depleted or SL-insensitive Arabidopsis mutants in the adult stage are drought stress hypersensitive and lack correct physiological and molecular responses to ABA (Ha *et al.*, 2014), while *max2* (SL-insensitive) mutants are hypersensitive to ABA at the seedling stage (Bu *et al.*, 2014). The SL–ABA relationship seems to be organ dependent: *Lotus japonicus* and tomato SL biosynthetic mutants show a decrease in the drought stress-induced ABA surge in leaves, suggesting a positive interaction (Liu *et al.*, 2015). In contrast, in Lotus roots, treatment with GR24 inhibits the osmotic stress-triggered increase of ABA concentration (Liu *et al.*, 2015), and drought stress decreases SL and increases ABA concentration in non-mycorrhizal roots of Lotus, tomato, and lettuce (Liu *et al.*, 2015; Ruiz-Lozano *et al.*, 2016), as would be the case for a negative interaction. Clearly, the interactions at the biosynthetic, catabolic, membrane transport, and signalling levels may be intricate and diverse in the different plant organs.

Although our results strongly suggest that GR24 affected sugar and anthocyanin accumulation through modulation of ABA concentration, other possibilities exist. Lv *et al.* (2018) recently showed that in Arabidopsis leaves GR24 induces stomatal closure also in ABA-depleted mutants, and that this ABA-independent effect could be triggered by an oxidative burst. A transcriptomic study suggested that an oxidative burst takes place at véraison in grape berries (Pilati *et al.*, 2007), and this could represent an additional mechanism of action of GR24 in grape berries.

### GR24 controls the expression of ABA metabolic but not of biosynthetic genes

We observed that the GR24 treatment significantly reduced ABA concentration in ABA-treated berries, compared with those treated with ABA only. The concentration of ABA is regulated by its biosynthesis, controlled by *NCED* genes, and by catabolism, which can follow both oxidation and conjugation pathways (Nambara and Marion-Poll, 2005). Oxidation reactions are catalysed by cytochrome P450 monooxygenases such as *ABA* 8'-hydroxylase (*CYP707A* gene family; Kushiro *et al.*, 2004; Saito *et al.*, 2004). In grapevine, three members of this gene family are described, among which *VvHYD1* and *VvHYD2* are most expressed in root and leaf (Speirs *et al.*, 2013). ABA oxidation to inactive compounds controls the drop in ABA concentration observed in leaves upon rehydration (Okamoto *et al.*, 2009) and in seeds upon imbibition (Okamoto *et al.*, 2006). ABA conjugation to ABA-glucose ester is performed by *ABA-GlucosylTransferase* (*AGT*) (Xu *et al.*, 2002). The grapevine homologue *VvGT1* is down-regulated after véraison (Sun *et al.*, 2015). In Arabidopsis, ABA-glucose ester is hydrolysed by a β-glucosidase (*BG1*) (Lee *et al.*, 2006). The grapevine homologue of this gene (*VvBG1*) was biochemically characterized and is up-regulated in berries at véraison (Sun *et al.*, 2015).

A straightforward hypothesis to explain the lower ABA concentration following GR24 co-treatment of ABA-treated berries is the activation of ABA catabolism. *CYP707A* genes are transcriptionally up-regulated following ABA treatment, suggesting an active contribution to homeostasis of free ABA levels (Cutler and Krochko, 1999; Saito *et al.*, 2004). We correspondingly observed a marked peak of *VvHYD1* and *VvHYD2* expression following ABA treatment. In the *in vitro* experiment, this peak, observed 72 h after treatment, did not cause a significant reduction of ABA concentration thereafter, probably due either to the high ABA levels induced by the treatment or to a relatively low amount of cytosolic ABA, the potential substrate of the cytosolic *CYP707A* gene products. Most interestingly, co-treatment with GR24 induced a further, significant expression increase of both hydroxylases, which could have elevated the enzyme activity to levels sufficient to observe the decrease of ABA at later sampling times. This finding, considering that GR24 application activates *CYP707A1* expression and enhances germination of *Phelipanche ramosa* seeds (Lechat *et al.*, 2012), while Arabidopsis CYP707A3 is up-regulated by gibberellin and brassinolide (Saito *et al.*, 2004), suggests that this gene family may mediate several hormone interactions in plants.

The effect of GR24 treatment on ABA conjugation is less clear: we observed no significant changes in expression of *VvGT1* (encoding a conjugating enzyme), and an activation of *VvBG1* (encoding a de-conjugating enzyme) transcript concentration, which could represent a homeostatic control of free ABA levels induced by the increase of ABA hydroxylation observed upon GR24 treatment. However, as *VvBG1* is two orders of magnitude less expressed than *VvGT1*, the contribution of de-conjugation to free ABA levels might be negligible.

Members of the *NCED* gene family are considered to be the main control point of ABA biosynthesis in Arabidopsis (Nambara and Marion-Poll, 2005) and are activated by ABA in some ecotypes (Cheng *et al.*, 2002). A second possible mechanism underlying the effect of GR24 on ABA-treated berries could thus be due to changes in ABA-induced ABA biosynthesis rate, which could contribute to the rise in ABA concentration, particularly in the cytosolic compartment. Two *NCED* genes were cloned from grapevine, *NCED1* being the most expressed in berries (Deluc *et al.*, 2007; Wheeler *et al.*, 2009; Zhang *et al.*, 2009). However, while *VvNCED1* expression decreased throughout the experiments, it was not significantly affected by ABA, as previously observed in tomato (Thompson *et al.*, 2000), suggesting that GR24 does not lower the free ABA concentration in ABA-treated samples by inhibiting biosynthesis at the transcriptional level.

### Membrane transport of ABA is regulated by GR24

Besides direct effects on ABA concentration, GR24 could control the expression of ABA membrane transport genes (Boursiac *et al.*, 2013). In Arabidopsis, *ABCG40* controls ABA cellular uptake: it is expressed in leaves, roots, and seed, and its down-regulation dampens physiological responses to ABA (Kang *et al.*, 2010). The ABA-induced *ABCG25*, localized to the vasculature, and in the endosperm, mediates ATP-dependent extrusion of ABA (Kuromori *et al.*, 2010; Kang *et al.*, 2015). Expression of these transport genes may affect the concentration of cytosolic free ABA, which interacts with the cytosolic PYL/RCAR receptors (Park *et al.*, 2009). In the grape berry, ABA transport genes have not been studied in detail yet, while *PYL/RCAR* genes have been identified and are expressed in vegetative tissue and in berries (Li *et al.*, 2012). We observed an early (i.e. 72 h and 48 h after treatment in the *in vitro* and *in vivo* experiments, respectively), transient induction of *VvABCG25* and *VvABCG40* transcript levels following ABA treatment, which was abolished upon GR24 co-treatment. These changes suggest that the cellular/apoplastic ABA concentration ratio may be affected by GR24 in ABA-treated berry skins by a decrease of import coupled to an increase of export activity. Additionally, since *VvABCG25* is two orders of magnitude less expressed than *VvABCG40* with respect to the same housekeeping genes, the dampening of ABA import might contribute more than the decreased export, resulting in a lower free ABA cellular concentration in ABA- and GR24-treated berry skins, compared with those treated with ABA alone.

### Do natural SLs play a role in grape berry ripening?

SLs are carotenoid-derived hormones, whose core biosynthetic pathway is based on the carotenoid isomerase D27 (Dwarf27), the carotenoid cleavage dioxygenases CCD7 and CCD8, and the P450 monooxygenase MAX1 (More Axillary Growth1) (Ruyter-Spira *et al.*, 2013). They are mostly, though not exclusively, produced in roots, where they are detected in the nanomolar range, and are thought to be transported to the shoots, where their concentration may be two orders of magnitude lower (Liu *et al.*, 2015) and, for most plant species, below the detection threshold. Genetic evidence shows that they are active in above-ground organs at such low concentrations, controlling shoot-specific traits such as axillary bud development (Brewer *et al.*, 2013). Also, reproductive defects of plants compromised in SL biosynthesis or perception suggest a largely unexplored role in flower and fruit development for certain species, besides juvenile to reproductive phase transition (e.g. in tomato, kiwifruit, Lotus, tomato, and petunia) (Snowden *et al.*, 2005; Ledger *et al.*, 2010; Kohlen *et al.*, 2012; Liu *et al.*, 2013).

In the grape berry, DNA microarray data suggest that *VvCCD7*, *VvCCD8*, and *VvMAX1* are differentially expressed in green and ripening berries (Young *et al.*, 2012), as also shown in tomato fruit for *SlCCD7* (Vogel *et al.*, 2010) and in kiwifruit for *AcCCD7* and *AcCCD8* (Ledger *et al.*, 2010). A reported attempt to quantify expression of putative *VvCCD7* and *VvCCD8* in above-ground organs of grapevine was not successful (Lashbrooke *et al.*, 2013). We assessed expression of the same two genes in berry skins during berry development by RT–qPCR, and confirmed a very low relative transcript level (Supplementary Fig. S1). Interestingly, expression of both *VvCCD7* and *VvCCD8* tended to increase in the late stages of ripening, in correspondence with the known decrease in ABA concentration after véraison (Wheeler *et al.*, 2009). In grapevine, no data are available on SL profiles and concentration. It must be noted here that SLs are usually undetectable in the aerial part of plants, and indeed the transcripts of the biosynthetic genes we tested are 10- or even 100-fold less concentrated than in roots, where SLs are more massively produced, especially under phosphate deprivation (data not shown). These preliminary results open up the possibility that changes in SL concentration at véraison may play a regulatory role in grape berry ripening.

While we clearly observed that GR24 limits the ripening effects of exogenous ABA, we were able to detect only very limited, and not significant, effects of GR24 treatments on non-ABA-treated berries. These observations seem contradictory, it being apparently unrealistic that GR24 may have such powerful effects on the signal induced by exogenous ABA, and at the same time to be ineffective on the endogenous ABA signal. A possible reconciling hypothesis is that endogenous SL is only one of several control points of the ABA concentration and/or signalling pathway, possibly co-operating at the molecular level with other effectors. In such a situation, additional, exogenous SL would not further affect the ABA signal in the absence of an increase of such co-operating effectors. It is well demonstrated that ABA can reinforce its own signal by ABA-dependent up-regulation of biosynthetic and signalling genes (Yang and Tan, 2014). Thus ABA treatment could entail an increase in expression of SL-co-operating molecular effectors, finally allowing exogenous SLs to interact with them to control the exogenous ABA concentration and signal.

## Supplementary data

Supplementary data are available at *JXB* online.

Table S1. Oligonucleotides used in this study for RT–PCR analysis

Fig. S1. Expression profiles of *VvCCD7* and of *VvCCD8* in skins of untreated *V. vinifera* during berry development

Supplementary Figure Table

## Abbreviations

ABA: abscisic acid
ABCG: ABC Transporter G Family Protein
PYL/RCAR: PYR-like/Regulatory Component of ABA Receptor
SL: strigolactone.
