# Supplementary material for: Exogenous strigolactone interacts with abscisic acid-mediated accumulation of anthocyanins in grapevine berries
Source: J Exp Bot. 2018 Jan 31;69(9):2391–401. doi: 10.1093/jxb/ery033 (PMC5913642; doi:10.1093/jxb/ery033)

Supplementary material

Table S1. **Oligonucleotides used in this study for RT-qPCR analysis**

Name	Gene accession (Grape Genome Database 12X V1)		Primer sequence (5'-3')
VvACT1	VIT_04s0044g00580	F<br>R	GCCCCTCGTCTGTGACAATG<br>CCTTGGCCGACCCACAATA
VvABCG25	VIT_18s0072g01220	F<br>R	ACTCTGTATTCGCCTTCCCC<br>GGGCATGTCTCCAACGATTC
VvABCG40	VIT_09s0002g05600	F<br>R	GCTAAGTTCTTCTGGTATCT<br>TTTGATTTGGTGTGGCAGC
VvBG1	VIT_01s0011g00760	F<br>R	TGATGGCCCCGGGAAAATAA<br>CCTGTCACCAAACTGCTGAA
VvCCD7	VIT_15s0021g02190	F<br>R	TGGGTATTTGAGGGCTTTTG<br>CCACCTTCTTCCCTCCTTTC
VvCCD8	VIT_04s0008g03380	F<br>R	GCTCAGGCTTCACAATCTCC<br>TAGTGAGGGTGTTGGGGAAG
VvHYD1	VIT_18s0001g10500	F<br>R	ATGGACTTCCAGCCAGATTG<br>GGACATCTCTCCAACCCAGA
VvGT1	VIT_03s0063g00050	F<br>R	CAAATGGGGAAGAAGGCGTG<br>CAGGCCTGCTCATCAATGGA
VvHYD2	VIT_02s0087g00710	F<br>R	TATTCAGTATGGCCCTTTTGCT<br>TTGATTGGTGGCACTGAGAG
VvMybA1	VIT_02s0033g00410	F<br>R	TAGTCACCACTTCAAAAAGG<br>GAATGTGTTTGGGGTTTATC
VvNCED1	VIT_19s0093g00550	F<br>R	GGTGGTGAGCCTCTGTTCCT<br>CTGTAAATTCGTGGCGTTCACT
VvUBI	VIT_16s0098g01190	F<br>R	TCTGAGGCTTCGTGGTGGTA<br>AGGCGTGCATAACATTTGCG
VvUFGT	VIT_16s0039g02230	F<br>R	CCCGGAATGTCTAAAGTACGTTT<br>AGCGAGTTTAGGTTTCCGAACA

Fig. S1. **Expression profiles of *VvCCD7* and of *VvCCD8* in skins of untreated *V. vinifera* during berry development**. Arrow shows time of ripening start (véraison). Bars are standard errors of the means.

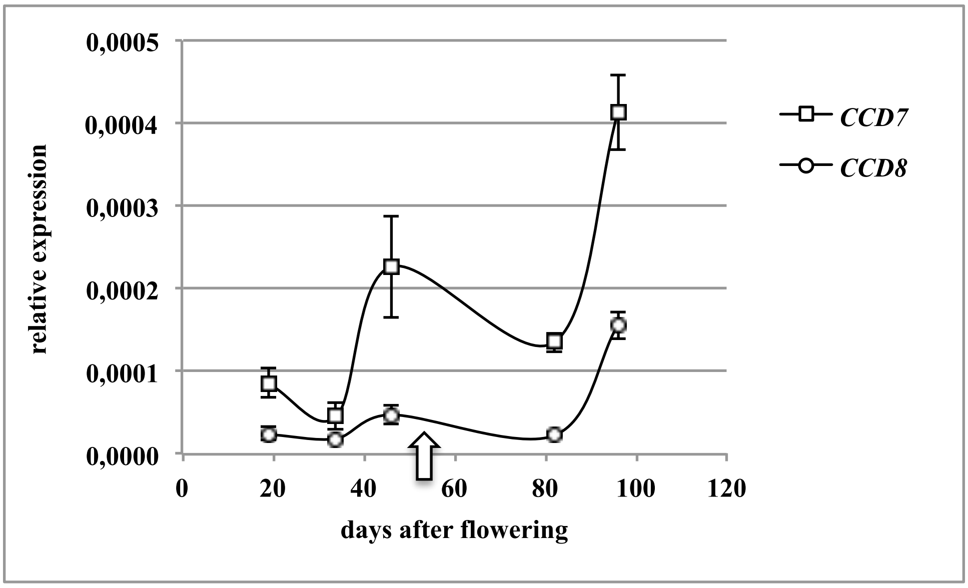

Supplement: Supplementary Figure Table [file ery033_suppl_supplementary_figure_s1_table_s1.pdf]
